# Management of acute pancreatitis in the “no man’s land”

**DOI:** 10.1007/s11739-025-03916-4

**Published:** 2025-04-06

**Authors:** Antonio Amodio, Nicolò de Pretis, Giulia De Marchi, Pietro Campagnola, Salvatore Crucillà, Federico Caldart, Luca Frulloni

**Affiliations:** University of Verona, Verona, Italy

**Keywords:** Acute pancreatitis, Correct diagnosis, Aetiology, Therapy

## Abstract

Acute pancreatitis (AP) is an inflammatory disease that can represent a challenge for clinicians, in fact, the early determination of its severity in the first 72 h is crucial for prognosis, recognizing the etiology and carrying out risk stratification to determine a more specific therapy. No accurate early prognostic scores for disease severity have been published, so the severity of AP often cannot be properly defined in the first few hours of the disease. This initial phase represents a "no man's land", in which there is no certainty in the stratification of the damage, prognosis is difficult to establish, therapy must be started promptly, although there is still no effective medical therapy against pancreatic enzymatic activation. Therefore, it is very difficult at this stage to make the correct decisions to achieve the best outcome for the patient with AP. Literature search was carried out using the PubMed database by entering early management of acute pancreatitis [title] or therapy of acute pancreatitis [title] and selecting the most relevant articles for the diagnosis and therapy of acute pancreatitis in clinical practice. This document provides suggestions on managing the key clinical decisions for patients suffering from AP before disease severity is defined, to achieve the best outcomes for patients with AP.

## Introduction

Acute pancreatitis (AP) is an inflammatory disease of the pancreas with a substantial impact on public health, with an estimated annual cost of 2.6 billion dollars in the United States[1]. Indeed, pancreatitis represents the 3rd most common principal diagnosis among gastrointestinal, liver, and pancreatic diseases in US hospitals in 2018 [2].

The estimated incidence of AP ranges from 13 to 45 per 100,000 inhabitants worldwide [3], from 4.6 to 100 per 100,000 inhabitants in Europe [4] and 32 per 100,000 inhabitants in Italy [5]. The crude rate of AP deaths was estimated to be approximately 1 per 100,000 deaths [2, 5].

AP is a heterogeneous disease that has a mild clinical outcome in the majority of cases (from 65 to 75%), with early patient discharge; a very low mortality rate, generally related to comorbidities; and low morbidity. The disease is clinically severe in the rest of patients, who require longer hospitalization, frequent admission to intensive care units, and radiological and endoscopic therapies to manage the disease’s complications, resulting in a substantial mortality rate (in approximately 4% of AP cases)[6]. The direct and indirect costs of pancreatitis are generally considerable but are particularly high for the severe form.

Early determination of AP severity is crucial for patient management and prognosis. In particular, the first 72 h after the onset of pancreatic pain are a crucial period for defining the disease severity, recognizing the aetiology and performing risk stratification to determine a more specific therapy. Different guidelines have considered these aspects, including those of the American Gastroenterological Association (AGA)[7], the American College of Gastroenterology (ACG)[8], the International Association of Pancreatology (IAP), the American Pancreatic Association (APA)[9], and the Italian guidelines for severe acute pancreatitis[10].

The early stage of acute pancreatitis is the most delicate period for patient management since in the clinical setting, AP may suddenly evolve towards a more severe necrotic form with multiorgan failure and the need for intensive care. However, no accurate prognostic score for disease severity has been published. Evaluation of severity requires 48–72 h to obtain an accurate definition of the presence and extension of pancreatic necrosis. Furthermore, even the presence of transient or persistent multiorgan failure requires 48–72 h to be accurately evaluated.

Therefore, the main relevant clinical problem is the patient’s management in the first 72 h, during which the severity of AP cannot yet be accurately defined. In this *“no man’s land”*, it may be difficult to make the right decision, particularly in nonskilled centres for pancreatic diseases. The present paper provides suggestions for managing the main clinical decisions for patients suffering from AP before the severity of the disease is determined. To identify studies relevant to the first 72 h, we conducted a systematic search of PubMed, EMBASE, and the Cochrane Library using the keywords “acute pancreatitis”, “early management”, “first 72 h”, “fluid resuscitation”, “enteral nutrition”, “severity prediction”. We included studies that assessed interventions initiated within 72 h of symptom onset, with a focus on those evaluating the effectiveness of early management strategies.

## Diagnosis of acute pancreatitis

The diagnosis of AP requires the presence of at least 2 of the following criteria, according to the Atlanta-revised classification [11]: 1. typical abdominal pain, 2. increased pancreatic enzymes greater than three times the normal limit, and 3. characteristic pancreatic inflammatory signs on abdominal imaging. The pain is typically constant, intense, and localized in the epigastrium, radiating to the right and left upper quadrant and to the back. Serum pancreatic amylase and/or lipase are universally used for diagnosis. Although pain and elevation of serum pancreatic enzymes are diagnostic criteria, none of them correlates with the severity of the disease; therefore, they do not have prognostic value. Accurate pain assessment is important since nonspecific abdominal pain in addition to a nonspecific increase in serum pancreatic enzymes or chronic pancreatic hyperenzymemia can lead to a misdiagnosis of acute pancreatitis. A cramp-like abdominal pain, associated with bowel movements, heartburn or meteorism, which responds to antispasmodic or Proton Pump Inhibitors is highly likely to be a pain of intestinal origin (e.g., gastric, colonic) and therefore cannot be categorized as pancreatic. Furthermore, pancreatic hyperenzymemia has been observed in perforated bowel, mesenteric infarction, intestinal obstruction, appendicitis, peritonitis, aortic aneurysm, ovarian cysts, and salpingitis [12, 13]. Imaging modalities (ultrasonography [US], computed tomography [CT], and magnetic resonance imaging [MRI]) are used to confirm the diagnosis only in patients with atypical abdominal pain or mild elevation of serum pancreatic enzymes. Imaging modalities (CT and MRI) are also useful for staging disease severity and evaluating the complications of the disease. Contrast-enhanced CT (CE-CT) can evaluate the extension of necrosis, which correlates with patient prognosis [14]. It is performed 48–72 h after the onset of pain rather than at the time of admission to the hospital [9]. MRI with cholangiopancreatography sequences has high sensitivity (95%) and specificity (97%) to diagnose common bile duct lithiasis [15] and to evaluate the contents of pseudocysts and walled-off necrosis (liquid or solid)[16, 17].

## Aetiology of acute pancreatitis

At first evaluation of the patient with AP it’s important to identify the aetiology of the inflammatory process, to use the specific therapy and, later, to prevent the recurrence of the disease. Clinical history is relevant to evaluate the presence of risk factors (alcohol and smoking), a familial cluster, the presence of previous episodes of AP, previous diagnosis of hypertriglyceridemia, autoimmune or vasculitic disorders [9]. Gallstones and alcohol are the most common causes of AP, representing together about 70% of all causes. ALT level is the most clinically useful parameter to achieve the diagnosis of biliary acute pancreatitis: with ALT levels greater than or equal to 150 IU/L, the probability of gallstone pancreatitis is 95% [18–20]. The diagnosis of acute biliary pancreatitis implies also to understand whether the stone has passed in the duodenum. The persistence of elevated serum ALT and direct bilirubin levels raises the suspicion of a stone in the common bile duct, whereas a rapid decrease/normalization supports the hypothesis of a passage of the calculus in the duodenum. Abdominal US evaluating biliary system is suggested to evaluate the biliary three and gallbladder. In doubtful cases, MRCP or EUS may be helpful before performing endoscopic retrograde cholangio-pancreatography (ERCP) to remove biliary stones [21]. Elevated alcohol intake and cigarette smoking, particularly in middle-aged men, raise the suspicion of paraduodenal pancreatitis, previously called groove pancreatitis or cystic dystrophy of the duodenal wall, a form of pancreatitis strongly linked to alcohol and cigarette smoking abuse. Symptoms related to duodenal wall inflammation are generally pancreatic-type pain, generally persistent requiring significant pain relief therapy up to morphine, significant weight loss, obstructive jaundice, duodenal occlusion. CE-CT or MRI-MRCP allow to make the diagnosis of this form of the disease in middle-aged man, with incorrect smoking and alcohol habits [22]. Pancreaticoduodenectomy seems to be a reasonable treatment choice in the setting of paraduodenal pancreatitis in consideration of the removal of the inflammatory mass responsible for local complications (obstruction of the pancreatic duct, jaundice, duodenal obstruction) [23]. Hypertriglyceridemia accounts for 3.4–5% of all causes of AP [24, 25]. Serum triglyceride levels (TG) should therefore always be performed in patients with AP in the emergency room or in the early hours after admission. TG, in fact, decrease rapidly over time above all because the patient is fasted after admission. For this reason, probably this aetiology is underestimated. The diagnosis is definitive if TG > 1000 mg/dl, probable if > 500 and < 1000, or possible if < 500 [26, 27]. Two meta-analyses showed that elevated serum TG levels resulted in worse AP outcomes [28, 29]. However, in a large, prospective, international cohort of patients, comparing hypertriglyceridemia-induced versus all other etiologies of AP, no significant difference was noted in terms of severity according to revised Atlanta Classification criteria, length of hospital stay, organ failure, or mortality [24]. Autoimmune pancreatitis (AIP) is a peculiar form of the disease that can start with an episode of AP. Imaging modalities (CT, MRI-MRCP, EUS) can easily diagnose AIP in diffuse form with typical findings, following international diagnostic criteria (ICDC) for AIP (level 1, parenchymal and ductal imaging)[30]. Pancreatic biopsies with forward-acquiring needles can be performed only in the presence of a focal involvement of the pancreas (level 2 ICDC) if a diagnosis of pancreatic cancer cannot safely be excluded [31]. Other various aetiologies can be determined not in the first hours, as they require further diagnostic investigations during hospitalization, such us gene mutation, pancreatitis associated with anatomic malformation of the ductal system, obstruction by solid lesions or IPMN, drugs, vasculitis, trauma.

## Assessment of severity of acute pancreatitis

Staging the severity of AP is essential for determining the best patient management to improve the clinical outcome. Administration of a correct and adequate therapy in the first 24 h after admission prevents local and systemic complications that can lead to the patient's death[21]. AP is an inflammatory process that may involve other organs, with the onset of cardiovascular, respiratory, and/or renal failure. The failure may be transient or persistent. Multiple and persistent organ failure have been associated with the morbidity and mortality of AP[32, 33]. Indeed, the duration of organ failure (OF) during the first week is closely related to the risk of local complications but mainly patient death. Patients with transient OF (lasting < 48 h) had lower mortality rates and fewer local complications than those with persistent OF (lasting > 48 h) (1% vs. 35% and 29% vs. 77%, respectively)[34]. The presence of OF can be evaluated by the Marshall score [35] (Table 1). Persistent organ dysfunction despite adequate fluid resuscitation requires continuous monitoring of vital signs in high-dependency care units and is an indication for ICU admission [32]. In fact, invasive ventilation becomes mandatory to correct dyspnea through mechanical ventilation if the oxygen supply or continuous positive airway pressure becomes ineffective. Furthermore, constant monitoring is essential to be able to achieve a balance between the benefit of the drugs used and avoid dangerous side effects, such as fluid overload, excessive sedation, and worsening of intra-abdominal pressure. Therefore, the best systemic support is guaranteed only in intensive care. Several scales and parameters have been proposed as prognostic scores, including serum levels of C-reactive protein (CRP) > 150 mg/L within the first 48 h, APACHE II > 8 during the first 24 h, or persistent OF after 48 h of hospitalization [21, 36]. Other evaluation systems validated over the years are the Ranson criteria, the Glasgow score, the Simplified Acute Physiology Score (SAPS II), the Sequential Organ Failure Assessment (SOFA), Bedside Index of Severity in Acute Pancreatitis Score (BISAP). Most scores are based on patient demographics, clinical characteristics, laboratory parameters or imaging modalities, and are assessed upon admission or within 48 h. There is no “gold standard” prognostic score to predict severe AP. The Bedside Index of Severity of Acute Pancreatitis (BISAP) score is one of the most applicable in clinical practice thanks to its simplicity, it is composed of the evaluation of five variables: Glasgow Coma Scale GCS < 15, SIRS, blood urea nitrogen levels > 25 mg/dL, age > 60 years, and pleural effusion on imaging [37]. In a recent meta-analysis, Capurso et al. retrieved data from 43 studies conducted on > 14,000 AP patients to investigate the accuracy of BISAP, APACHE‐II, Ranson, and SIRS in predicting SAP. The main finding of the study is that all scoring systems have modest scoring and limited clinical utility, as the actual post‐test probability of SAP never reached 50% when scores were predicting a severe course and ranged between 5 and 12% when they were predicting a non‐severe course [38]. The utility of the previously described scoring systems for predicting SAP is significantly limited by several critical issues. One of the primary concerns is their poor accuracy, which renders their performance in clinical practice essentially equivalent to a coin toss. Moreover, the applicability of these scoring systems is further constrained by their development and validation in specific patient populations, reducing their ability to accurately predict outcomes in individual patients.

**Table 1:** Modified Marshall Scoring System for Organ Dysfunction

	Score^a^				
Organ system	0	1	2	3	4
Respiratory *(PaO*_*2*_*/FIO*_*2*_*)*^***b***^	> 400	301–400	201–300	101–200	< 101
Kidney *(serum creatinine, μmol/L)*	< 134	134–169	170–310	311–439	> 439
Kidney *(serum creatinine, mg/dL)*	< 1.4	1.4–1.8	1.9–3.6	3.7–4.9	> 4.9
Cardiovascular *(systolic blood pressure, mm/Hg)*	> 90	< 90 fluid responsive	< 90 No fluid responsive	< 90 pH < 7.3	< 90 pH < 7.2

*FIO*_*2*_ fraction of inspired oxygen, *PAO2* partial pressure of arterial oxygen

^a^Score ≥ 2 for any system defines the presence of organ failure

^b^for non ventilated patients, FIO2 can be estimated by the rate of supplemental oxygen (room air = 21%, 2L/min = 25%, 4L/min = 30%, 6–8L/min = 40%, 9–10/min = 50%)

Increased intra-abdominal pressure (IAP) and BMI show greater sensitivity and specificity compared to the commonly used severity rating systems [32, 33, 39]. Recently, the introduction of the Harmless Acute Pancreatitis Score (HAPS) has proven to be a valid tool for identifying cases of acute pancreatitis characterized by a non-severe course [40]. HAPS is an easily accessible and efficient scoring algorithm that rapidly identifies patients who may experience non-severe progression of AP. In particular, the assessment using HAPS can be completed within just an hour of admission, highlighting its rapid applicability. This discrepancy in timing compared to classic scoring systems highlights the advantage of HAPS in providing a timely and effective assessment, which is critical for rapid decision-making in the clinical management of acute pancreatitis.

From a radiological point of view, the assessment of the severity of AP can be determined by Mortelè Modified CTSI Scoring [41], which is more accurate than Balthazar CTSI Scoring[42]. The Balthazar CTSI is calculated by adding the points related to the CT findings and the points referring to the extent of necrosis. As shown in Table 2, the modified CTSI is calculated by summing the evaluated parameters, and the total score is then categorized as mild (0–2 points), moderate (4–6 points), or severe pancreatitis (8–10 points). Additionally, 2 points are added if extrapancreatic findings are present.

**Table 2:** Modified CT severity score [41]

Prognostic indicator	Points
Pancreas inflammation	
Normal pancreas	0
Intrinsic pancreatic abnormalities ± inflammatory changes in peripancreatic fat	2
Pancreatic or peripancreatic fluid collection or peripancreatic necrosis	4
Pancreatic necrosis	
none	0
≤30%	2
> 30%	4
Extrapancreatic complications (pleural effusion, ascites, vascular complications, parenchymal complications or gastrointestinal tract involvement)	2

The severity of AP is categorized as mild (0–2 points), moderate (4–6 points), or severe (8–10 points)

Since multiple factors interact in a nonlinear and complex manner in determining the actual risk of developing SAP, artificial intelligence algorithms could represent an appropriate tool to improve predictive capability. It is therefore likely that artificial intelligence will become an increasingly widespread approach to achieving early and accurate prediction of the severity of acute pancreatitis, overcoming the limitations of currently available scoring systems.

## Therapy of acute pancreatitis

No specific pharmacological therapy for the treatment of AP is currently well established[43], observational studies and randomized controlled trials have established best practices leading to a reduction in morbidity and mortality in AP. The basic treatment of patients with AP consists of providing adequate resuscitation with isotonic intravenous fluids, pain control, and constant monitoring of vital signs and organ function[32].

### Fluid resuscitation

Intravenous fluids should be set up to promote pancreatic microcirculation, prevent pancreatic necrosis and improve outcomes, the volume of which should be assessed based on the patient's weight, pre-existing cardiac and renal disease[21, 32, 44]. Fluid resuscitation should be started before imaging at admission. Laboratory indicators of adequate volemia to be monitored are haematocrit, creatinine and urea nitrogen, and blood lactate[32]. Fluid resuscitation remains central to early management, but the type and rate of fluids are debated. A randomized controlled trial (RCT) performed by Wu et al.[45], documents a greater benefit of Ringer lactate compared to normal saline due to the anti-inflammatory effect and better regulation of the potassium level. Ringer's lactate fluid resuscitation provides an 84% reduction in the incidence of SIRS in patients and causes a marked decrease in CRP (from 104 mg/dL to 54 mg/dL). The WSES 2019 guidelines suggest isotonic crystalloids be the preferred fluid[32]. Since a very low quality of evidence due to a lack of RCT evidence addressing the optimal initial rate, volume and duration of AP fluid resuscitation, the 2018 AGA guidelines did not provide specific recommendations for fluid management, suggesting a goal-directed therapy, defined as the titration of intravenous fluids to specific clinical and biochemical targets of perfusion (e.g., heart rate, mean arterial pressure, haematocrit, central venous pressure, urine output, blood urea nitrogen concentration)[46]. ESGE guidelines give more specific advice by recommending initial targeted intravenous fluids therapy with lactated Ringer’s (e.g. 5–10 mL/kg/h) at the start of pancreatitis[47]. In a recent multicenter randomized study by de-Madaria et al., early aggressive fluid resuscitation with Ringer’s solution (20 mL/kg bolus, followed by 3 mL/kg/h) resulted in a higher incidence of fluid overload without improvement in clinical outcomes compared to patients with moderate resuscitation (10 mL/kg bolus in hypovolaemic patients or no bolus in normovolemic patients, followed by 1.5 mL/kg/h), specifically 20.5% vs 6.3%[48].

### Nutritional support

*“Put the pancreas at rest”* is the dogma for therapy in the past, since it was believed that complete fasting reduced activation of digestive enzymes and, consequently, the inflammatory process. However, early refeeding has been suggested in mild acute pancreatitis since the last century[49]. Recent RCTs show that early oral/enteral feeding in AP patients causes no adverse effects and leads to reductions in pain, opioid use, and length of stay[21]. Intestinal barrier failure facilitates the passage of bacteria (*“bacterial translocation”*) and inflammatory products through the intestinal wall to pancreatic necrotic collections. Early refeeding promotes the integrity of the gut barrier reducing gut permeability and preventing bacterial translocation and its complications[50]. Enteral nutrition reduces oxidative stress and systemic exposure to endotoxins and reduces the risk of clinically severe forms[12, 32, 36]. Therefore, some Authors suggested that oral nutrition must be started early with no dietary restriction in patients with mild pancreatitis, reducing hospitalization times[51, 52]. However, in a multicenter RCT with patients with predicted severe AP, attempted oral refeeding three days after pain onset was tolerated in 69% of patients[53]. While severity stratification may require 48–72 h, early oral feeding (within 24–48 h) is recommended unless contraindicated (e.g., ileus, vomiting). Studies demonstrate that early enteral nutrition reduces infectious complications, mortality, and hospital stays compared to delayed refeeding. Several guidelines for AP recommend enteral nutrition over total parenteral nutrition (TPN)[7, 9] for a more favourable outcome, such as fewer infectious complications, surgical interventions, and mortality[54]. In conclusion, early refeeding is strongly suggested in mild AP. If severe form is suspected, an oral diet can be started unless contraindicated and is useful for improving the patient’s outcome. Enteral nutrition should be offered in severe forms and if oral refeeding has failed. TPN use in AP must be discouraged and used only if oral refeeding and enteral refeeding have failed or unavailable.

### Pharmacological therapy

No specific pharmacological therapy has proved effective in the early treatment of pancreatitis[32]. Randomized trials have shown no clinically benefit in the management of AP with anti-inflammatory agents, antiproteases (e.g. gabexate) and antisecretory agents (e.g. octreotide) [55–57]. Antibiotics do not reduce the risk of infected necrosis and do not improve morbidity e mortality in patients with severe AP[58]. Guidelines do not recommend the routine use of prophylactic antibiotics in patients with AP[9, 32, 47] Antibiotics should be reserved for confirmed infections rather than prophylactic use in the first 72 h. During this early phase, systemic inflammatory response syndrome (SIRS) is common, but true infections are rare (< 5% of cases), and prophylactic antibiotics increase the risks of fungal superinfections[7]. In cases of biliary pancreatitis, for example, there may also be acute cholangitis or acute cholecystitis. In this setting, indeed, a concomitant elevation of hepatocytolysis and cholestasis indices, an increase in procalcitonin and imaging showing dilation of the biliary tract with biliary obstruction (e.g. due to common bile duct stones) may justify the early use of antibiotics for sepsis control. The routine use of proton pump inhibitors is not recommended in patients with severe acute pancreatitis[10]. A recent metanalysis shows no influence of PPI on the clinical course of AP[59]. Moreover, PPI use in the setting of severe AP is associated with the occurrence of greater duodenal bacterial overgrowth and candida infections than in the not-treated group[60]. There is no consensus on the treatment of pain in AP both for the type of drug and for route of administration. There are only RCTs studies including a few patients with weak results[61]. Nonsteroidal anti-inflammatory drugs (NSAIDs) are often used, although they often contribute to intestinal damage and kidney failure. A systematic review concluded that opioids could reduce the need for supplemental analgesics without increasing adverse effects[62]. Later in 2020, Kumar et al. documented that both diclofenac and tramadol are equally effective in pain control in AP [63]. In 2021, Cai et al. published an updated meta-analysis with additional RCT studies, showing opioids to be superior to non-opioid analgesics[64]. However, a recent systematic review showed the absence of a superior role of opioids compared to the control group in the population in terms of adverse effects, pain severity, use of additional drugs, length of hospital stay and mortality [65]. Concerns about opioid use include the spasm of the sphincter of Oddi, respiratory depression, paralytic ileus, and chronic abuse. These adverse events have limited the opioid’s prescription in AP, although such events are not reported as significant in meta-analysis. Epidural administration appears effective within the first 24 h of AP although featured in only two RCT [66, 67]. It should be emphasized that there is a paucity of level 1 evidence to guide pain management in acute pancreatitis [68]. Therefore, further studies with more solid evidence are required to implement the management of pain control in AP.

The potential benefits and risks of NSAIDs, including chronic use of Acetylsalicylic acid, in patients with AP are poorly understood and the studies conducted have produced conflicting results. Several retrospective studies reported an association between NSAIDs and reduced pancreatic necrosis, organ failure and in-hospital mortality [69, 70]. Vutipongsatorn et al. documented that the use of NSAIDs was not associated with a reduced risk of pancreatic necrosis[71]. Furthermore, a randomized controlled trial comparing 48-h rectal diclofenac and placebo found no significant differences between the two groups in the risk of OF and SIRS[72]. Finally, a recent retrospective multicenter study found no significant correlation between the use of nonsteroidal anti-inflammatory drugs (NSAIDs) around the onset of acute pancreatitis (AP) and the risk or severity of OF and SIRS during the initial week. This study raises doubts about the clinical efficacy of NSAIDs in mitigating OF in the case of AP[73]. Infliximab is a monoclonal antibody biologic drug used for the treatment of autoimmune diseases as it blocks the TNF-α activity. Currently, a double-blind, placebocontrolled, multicenter RCT (RAPID-I trial) designed to evaluate the effectiveness and safety of early infliximab initiation in the treatment of AP is ongoing[74]. It is necessary to identify a targeted treatment and explore new therapeutic approaches. Existing therapies are basically only supportive and do not target the etiology of inflammation in AP. One of the most important challenges that remains is determining the timing and frequency of pharmacological intervention. Unfortunately, despite progress in understanding the pathophysiological mechanisms of pancreatic inflammation, there are currently no proven effective therapies. A recent review indicates that available data including both randomized and non-randomised controlled trials in hypertriglyceridemia-induced acute pancreatitis show a notable degree of consistency, suggesting that plasma triglyceride exchange (TPE) leads to a moderately accelerated reduction in triglyceride levels, particularly within the first 24 h (about 70% compared to 50% with therapy conservative treatment including insulin) [75]. Furthermore, there are no definitive observational data on the beneficial effects of TPE on clinical outcomes. The authors therefore suggest that there is no role for TPE in non-severe HTG-AP cases and that insulin can be used in these cases, although clear evidence of clinical efficacy is lacking [76]. The results of the ongoing ELEPHANT study will clarify the role of medical therapy in hypertriglyceridemia-induced AP[77].

## Endoscopic treatment

Urgent ERCP (≤ 24 h) for biliary drainage is recommended in patients with AP combined with cholangitis. In patients with persistent biliary obstruction but no cholangitis, ERCP should be performed within 72 h[47]. In an RCT study in patients with predicted severe pancreatitis from gallstones but without cholangitis, urgent ERCP with sphincterotomy did not reduce the endpoint of major complications or mortality compared with conservative treatment. ERCP should not be performed in patients with acute biliary pancreatitis and neither cholangitis nor ongoing bile duct obstruction[78]. In cases of AP due to gallstone disease, cholecystectomy should be performed to prevent recurrent episodes of AP[79, 80].

## Discussion and conclusions

**Fig. 1:**
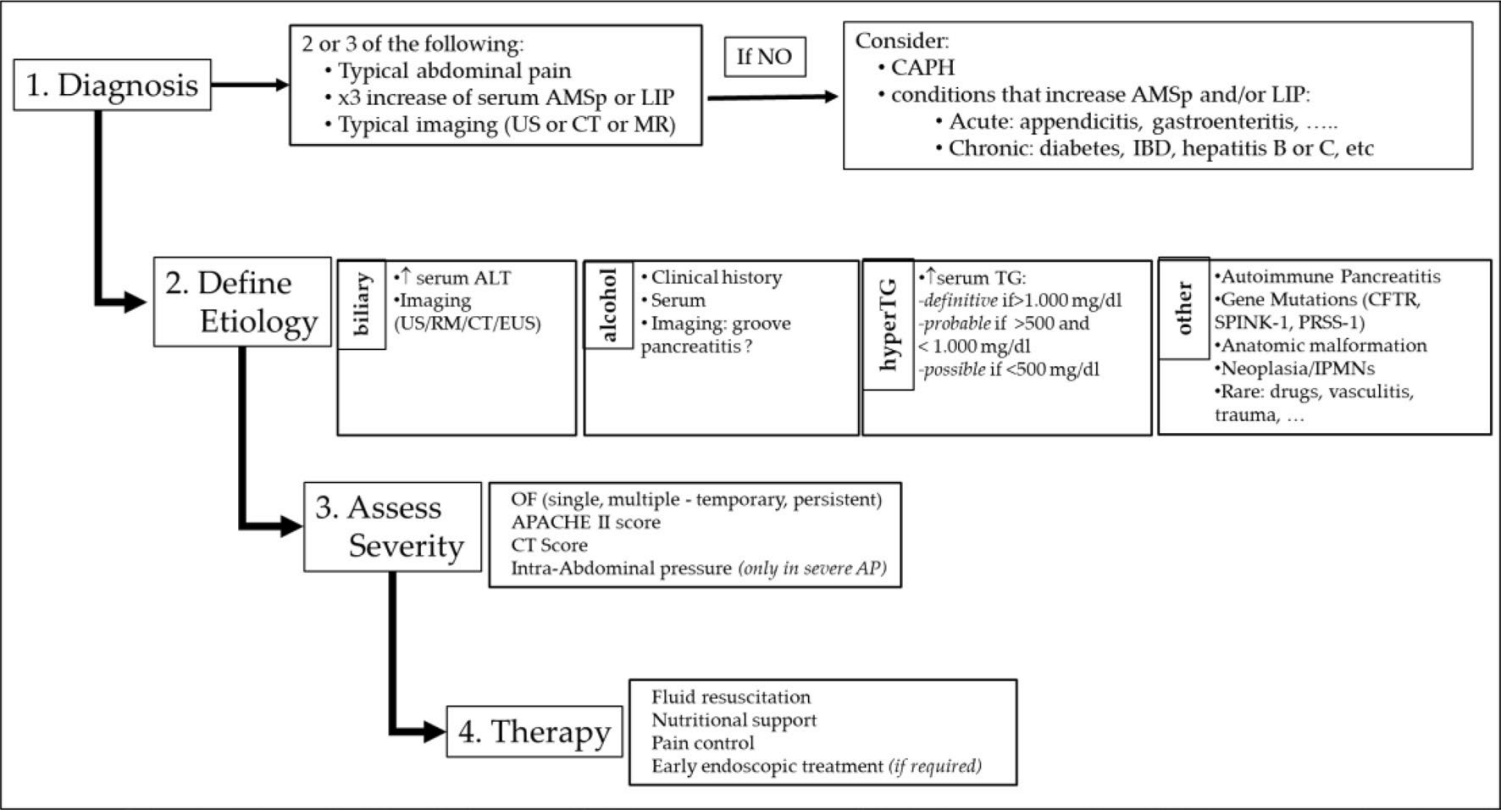
Flowchart for the management of Acute Pancreatitis. Perform a correct diagnosis of AP, considering the clinical context and alternative causes of pancreatic hyperenzymemia. Subsequently, define the etiology accurately. Stage the severity of pancreatic damage. Finally, establish supportive therapy. Abbreviation: AMSp = pancreatic amylase; LIP = Lipase; CAPH = Chronic asymptomatic pancreatic hyperenzymemia; IBD = Intestinal bowel disease; HyperTG = hypertriglyceridemia; US = Ultrasound; TC = Tomography computed; MR = magnetic resonance imaging; CFTR = Cystic fibrosis transmembrane conductance regulator; SPINK-1 = Serine protease inhibitor Kazal type 1; PRSS-1 = Cationic trypsinogen type 1; IPMN = Intraductal papillary mucinous neoplasm; OF = Organ failure

Acute pancreatitis represents a challenge for clinicians, especially in the first hours after onset. A correct clinical evaluation through the identification of the etiology, a correct staging of the severity, and an early adequate therapy are essential to try to avoid evolution towards a severe form. The clinical and instrumental assessments are often complex, and not always reliable in the first 24–72 h. Furthermore, all scoring systems have limited clinical utility, as the actual post‐test probability of SAP never reaches high performace when scores predicting a severe course. The Harmless Acute Pancreatitis Score (HAPS) is an easily and efficient scoring algorithm that rapidly identifies patients who may experience mild progression of AP, therefore, it could represent an additional tool for rapid decision-making in the early hours. No specific pharmacological therapy for the treatment of AP is currently well established, treatment consists of providing adequate moderate resuscitation with isotonic intravenous fluids, pain control through the use of opioids or NSAIDs, and constant monitoring of vital signs and organ function. Early refeeding is strongly recommended in mild AP, while if a severe form is suspected, an oral diet can be started unless contraindicated (e.g., ileus, vomiting), reserving enteral nutrition only in severe forms if oral refeeding has failed. The use of total parenteral nutrition in AP should be used only if oral refeeding and enteral refeeding have failed. Endoscopic biliary drainage in acute pancreatitis is indicated in cases of acute cholangitis (≤ 24 h) or within 72 h in cases of persistent biliary obstruction. Figure 1 shows a summary flowchart of the management to be followed in the first 72 h of AP.

In conclusion, in the first hours of AP patients need constant monitoring to evaluate the onset of persistent multiorgan failure, since acute pancreatitis is a potential systemic disease. Unfortunately in this setting, defined as *no man's land*, early and reliable predictive scores of severity are not yet available. Furthermore, one of the major challenges that remains to be addressed for effective management of AP is to determine a pharmacological therapy that counteracts the pathophysiological mechanisms of inflammation, currently limited to the role of support and control of systemic involvement. The first 72 h of acute pancreatitis demand a paradigm shift: from reactive to proactive care. Clinicians must prioritize goal-directed fluids, early enteral nutrition, and antibiotic stewardship while acknowledging the limitations of existing severity scores. Future research should focus on novel biomarkers for real-time risk stratification, including the application of Artificial Intelligence analytics, as well as standardized protocols for this critical time.

## Data Availability

No experimental data used for this review.
